# Cellular mechanisms linking cancers to obesity

**DOI:** 10.15698/cst2021.05.248

**Published:** 2021-04-12

**Authors:** Xiao-Zheng Liu, Line Pedersen, Nils Halberg

**Affiliations:** 1 Department of Biomedicine, University of Bergen, N-5020 Bergen, Norway.

**Keywords:** obesity, cancer, mouse models, adipokines, metabolism, inflammation, extracellular Matrix remodeling

## Abstract

Obesity is epidemiologically linked to 13 forms of cancer. The local and systemic obese environment is complex and likely affect tumors through multiple avenues. This includes modulation of cancer cell phenotypes and the composition of the tumor microenvironment. A molecular understanding of how obesity links to cancer holds promise for identifying candidate genes for targeted therapy for obese cancer patient. Herein, we review both the cell-autonomous and non-cell-autonomous mechanisms linking obesity and cancer as well as provide an overview of the mouse model systems applied to study this.

## INTRODUCTION

Obesity is defined as an excessive accumulation of adipose tissue, which stores energy in the form of triglycerides. This mainly occurs when caloric intake exceeds energy expenditure. Currently, 1.9 billion adults and 38 million children worldwide are categorized as overweight – numbers that have tripled since 1975 [1]. Dating back to the mid-1980's, clinicians reported that obese patients diagnosed with breast cancer have poorer survival outcomes [2]. Large epidemiological studies have since supported and expanded on these observations and estimates now suggest that obesity is implicated in 15-20% of cancer-related mortalities (reviewed in [3, 4]). This is a consequence of obesity's association with an increased risk of developing cancers (cancer incidence) and with specific obesity-linked biological effects on cancer progression in combination. Currently, obesity has been associated with 13 cancer types (reviewed in [5]). Epidemiological cross-comparisons have revealed that these interactions involve biological specificities. Examples include: i) sex-related correlations – the association between obesity and colon cancer are significantly more pronounced in males than in females, ii) specific histological subtypes – obesity is correlated with esophageal adenocarcinoma, but not esophageal squamous cell carcinoma and iii) menopause state – the risk of breast cancer is associated with obesity in postmenopause, but not in premenopause [6].

The obese environment is highly complex and comprises changes in serum levels of multiple circulating factors such as glucose, leptin, glucagon, adiponectin, glucagon-like-peptide-1, insulin, free fatty acids (FFA) and cholesterol; altered sleeping behavior; altered gut microbiome; altered pharmacodynamics of therapeutics, poor wound healing and post-operative infections, and the development of comorbid diseases (including chronic low-grade inflammation, heart disease, type 2 diabetes and hypertension). Such phenotypic complexity is present both systemically and in local tissue microenvironments. From the emerging molecular understanding of how such complexity in the obese environment links to cancers, we are beginning to uncover new and fascinating interactions between the physiological state of host and tumor behavior.

**Figure 1:**
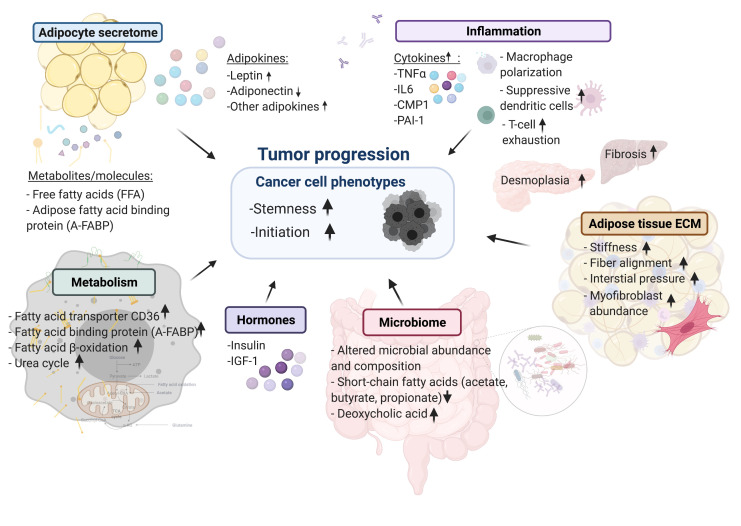
FIGURE 1: Summary of cellular mechanisms linking cancers to obesity.

To recapitulate the complex obesogenic environment and uncover the molecular mechanisms that link obesity and cancer the field has so far relied on mouse models. In this review, we provide an overview of these model systems as well as proposed molecular and cellular processes by which the obese environment affects tumor initiation and progression (summarized in **Figure 1**).

## ANIMAL MODELS OF THE OBESITY-CANCER CONNECTION

As *in vitro* models generally do not demonstrate the full complexity of the obesogenic phenotype, *in vivo* animal models have been essential research tools to address the mechanisms underlying the obesity-cancer connection. The most widely used models are either genetic or diet-induced obesity (DIO) models. Upon a cancer challenge these models broadly display increased tumor initiation and growth, relative to their non-obese counterparts. Importantly, these models often support the biological specificities of the epidemiological link between obesity and cancer. For example, consistent with human data, obesity leads to increased tumor burden and faster disease progression in oncogenic *Kras*-driven pancreatic ductal adenocarcinoma (PDAC), but not in lung cancer [7]. **Table 1** presents an overview that details how both genetic and DIO models have been applied to cancer research.

**TABLE 1.** Overview of *in vitro* and *in vivo* studies.

**Obesity model**	**Diet, duration**	**Cancer model**	**Obese tumor phenotype/pro-posed mechanism (keywords)**	**Ref**
**Breast cancer**				
DIO	HFD (60% kcal from fat), 8 weeks + special diet	T (MMTV-Wnt-1, 5×10^4 cells, orthotopic)	Calorie restriction and rapamycin inhibit mammary tumor growth in postmenopausal obesity	[120]
DIO	HFD (60% kcal from fat)	T (E0771/MDA-MB-231, 1×10^6 cells, orthotopic; LLC/ID8, 1×10^6 cells, subcutaneous)	Obesity-induced expanded adipose stromal cells promote tumor growth	[121]
DIO	Western diet (21% fat), 45 days	CICM (N-methylnitrosourea)	Obesity promotes cancer stemness phenotype via leptin-STAT3-G9a histone methyltransferase signaling axis	[40]
DIO	HFD (36% kcal from fat), 10 weeks	T (Py8119, 1×10^5 cells / E0771, 2×10^5 cells, orthotopic)	Obesity-associated NLRC4 inflammasome activation/interleukin (IL)-1 signalling promotes breast tumor growth and angiogenesis	[108]
DIO	HFD (60% kcal from fat), 15 weeks	T (86R2 or 99LN, 1.5×10^6 cells, orthotopic)/metastasis assay (99LN, 2×10^6 cells, tail vein)	Obesity-associated inflammation promotes breast cancer metastatic progression	[13]
DIO	HFD (60% kcal from fat), 13 weeks	Metastasis assay (metM-Wntlung, 2,5×10^3, tail vein)	HFD fed mice have reduced overall survival and higher incidence of lung macrometastases	[122]
DIO	HFD (60% kcal from fat), 5-6 months, GEMM (45% kcal from fat), 14 months	T (E0771, 5×10^5 cells + limiting dilution, orthotopic), GEMM (MMTV-TGFα)	A-FABP promotes tumor stemness and aggressiveness through activation of the IL-6/STAT3/ALDH1 pathway	[90]
DIO	HFD (60% kcal from fat), 9 - 11 weeks	T (E0771 or Py230, orthotopic)	Obesity induces hypoxia, neutrophil infiltration and EMT, leading to the faster growing tumors and an increase in metastasis-initiating cells	[103]
DIO	HFD (60% kcal from fat), 12 weeks or 4 days	T (E0771 (1×10^4 - 2×10^5 cells)/PY8119, (2,5 - 5×10^3 cells), orthotopic)	Metabolically activated adipose tissue macrophages link obesity to triple-negative breast cancer	[20]
DIO	HFD (60% kcal from fat), 12 weeks	T (E0771, 5×10^5 cells, orthotopic)	Heparanase regulates macrophage functions to promote tumor progression	[86]
DIO	HFD (60% kcal from fat), 5 weeks	T (E0771, 2 x 10^5 cells, orthotopic)	A non-canonical function of BMAL1 metabolically limits obesity-promoted triple-negative breast cancer	[95]
DIO	HFD (60% kcal from fat), 12 weeks	T (E0771, 5×10^5 cells, orthotopic)	Enhanced resistin secretion in obese mammary adipose issue via FFA/PPARγ/TAZ axis promote breast tumorigenesis	[64]
**Colon/small intestine cancer**				
DIO	HFD (56.7% kcal from fat), until end-point	CICM (azoxymethane)	Adiponectin supresses colorectal carcinogenesis under the HFD condition	[56]
DIO, ob/ob, db/db	HFD (56.7% kcal from fat), until end-point	CICM (azoxymethane)	Leptin acts as a growth factor for colorectal tumors at stages sub-suquent to tumor initiation	[32]
**Colon/small intestine cancer**				
DIO	HFD (40% kcal from fat), 8 weeks	GEMM (ApcMin/+)	HFD alter expression of inflammatory markers and increase tumorigenesis	[123]
DIO	HFD (60% kcal from fat), 22 weeks/until endpoint	GEMM (KrasG12Dint)	HFD mediated dysbiosis promotes carcinogenesis independently of obesity	[102]
DIO	HFD (60% kcal from fat), 9-14 months	-	HFD enhances stemness and tumorigenicity of intestinal progenitors	[12]
DIO	HFD (60% kcal from fat), 8 - 10 weeks	T (MC38, 1×10^5 cells, subcutaneous)	Fatty acid metabolism impair T cells infiltration and function and promote cancer growth	[92]
**Kidney cancer**				
DIO	HFD (60% kcal from fat), 20 weeks	T (RenCa-Luc, 2 × 10^5 cells, orthotopic)	HFD promotes dendritic cell infiltration, which suppress T cell expansion and enhanced tumor growth	[104]
DIO, ob/ob	HFD (60% kcal from fat), 20 weeks	T (CRL-2947-Luc, orthotopic)	Elevated leptin during diet-Induced obesity reduces the efficacy of tumor immunotherapy	[41]
**Liver cancer**				
DIO, ob/ob	HFD (59% kcal from fat)	CICM (Diethylnitrosamine)	Obesity induced low-grade inflammation promoted the hepatic procarcinogen DEN-induced HCC	[100]
DIO, ob/ob	HFD (60% kcal from fat), until endpoint	CICM (7,12-dimethylbenz(a)anthracene, DMBA)	Obesity-induced gut microbial metabolite promotes liver cancer through senescence secretome	[11]
DIO	HFD (59% kcal from fat), until endpoint	GEMM (MUP-uPA)	ER stress cooperates with hypernutrition to trigger TNF-dependent spontaneous HCC development	[101]
DIO	HFD (60% kcal from fat), until endpoint	CICM (7,12-dimethylbenz(a)anthracene, DMBA)	Gut microbiota promotes obesity-associated liver cancer through PGE2-mediated suppression of antitumor immunity	[124]
DIO	HFD (43% kcal from fat, 40 weeks)	Alb-Cre;Ptpn2fl/fl	Obesity drives STAT-3 dependent hepatocellular carcinoma	[82]
**Melanoma**				
DIO	HFD (60% kcal from fat)	T (B16,1*10^6, subcutaneous)	Paradoxical effects of obesity on T cell function during tumor progression and PD-1 checkpoint blockade	[109]
**Myeloma**				
DIO	HFD (60% kcal from fat), 15 weeks	Vk12598 (5×10^5, intrafemorally), 5TGM1 (1×10^6, intravenous)	Acetyl-CoA synthetase 2 - a critical linkage in obesity-induced tumorigenesis in myeloma	[125]
**Oral carcinomas**				
HFD	HFD (60% kcal from fat), 7 days	T (Detroit-562 cells, orthotopic,)	HFD is able to boost the metastatic potential of CD36+ metastasis-initiating cells to promote cancer metastasis	[88]
**Ovarian cancer**				
-	-	T (coinjection: SKOV3ip1 cells + human adipocytes, subcutaneous)	Adipocytes promote ovarian cancer metastasis and provide energy for rapid tumor growth	[89]
**Pancreas cancer**				
DIO	HFD (60% kcal from fat)	GEMM (KC, 48Cre-K-rasLSL-G12D/+)	Inflammation (TNFa signalling) and increased fatty acid mitochondrial beta-oxidation links obesity to tumor promotion	[99]
ob/ob, db/db	-	T (Pan02, 2,5*10^5, sub.c)	Altered adipokine milieu and insulin resistance promotes cancer growth and dissemination	[126]
DIO	HFD (60% kcal from fat), >30 days/until endpoint	GEMM (KC, KrasG12D, LSL-Kras/Ela-CreERT and LSL-Kras/PDX1-Cre mice)	Activation of Kras via COX2 leads to pancreatic inflammation and fibrosis and developement of PanINs and PDAC	[98]
DIO	HFCD (40% kcal from fat), 3 months	GEMM (KC, PDX1-Cre;LSL-KRASG12D)	Increase in inflammatory cells, cytokines, chemokines, and stromal fibrosis accelerates early pancreatic neoplasia	[127]
DIO	HFD (60% kcal from fat), 10 weeks	T (Pan02, AK4.4, graft, orthotopic)	PIGF/VEGFR-1 signaling promotes macrophage polarization and accelerated tumor progression in obesity	[97]
DIO, ob/ob	HFD (60% kcal from fat), 10 weeks	T (Pan02, AK4.4, graft, orthotopic)	Obesity-induced inflammation and desmoplasia promote pancreatic cancer progression and resistance to chemotherapy	[42]
DIO	HFD (60% kcal from fat), >50 days/until endpoint	GEMM (KC, KrasG12D/Cre) and T (cells from KPC model)	Depletion LCN2 diminishes ECM deposition, immune cell infiltraton, PanIN formation, and tumor growth	[128]
DIO	HFD (60% kcal from fat), 8 weeks	T (xenograft: 10^5 to 10^6 cells, syngeneic (KPC): 2,5-5*10^4, orthotopic), GEMM (KPC)	Mitochondrial arginase (ARG2) is induced upon obesity and scilencing or loss suppresses tumorigenesis	[94]
DIO	HFCD (40% kcal from fat), >3 months	GEMM (KC,P48+/Cre;LSL-KRASG12D)	HFCD increase cancer incidence, fibrosis and inflammation of KC mice in addition to reducing autophagic flux of PanIN lesions.	[129]
DIO	HFD (60% kcal from fat), 3 months	NA (CK19-RasV12-GFP)	Obesity suppresses cell-competition-mediated apical elimination of RasV12-transformed cells from epithelial tissues	[130]
DIO	HFD (61,6% kcal from fat), 10 weeks	GEMM (KC, KRASG12D/+)	HFD heightens aerobic glycolysis through hyperactivation of oncogenic KRAS	[93]
DIO	HFD (60% kcal from fat), 10 weeks	GEMM (KC, fElasCre-ERT;KrasLSL-G12D/+, Ptf1aCreERT;KrasLSL-G12D/+)	KRAS reduces expression of FGF21 in acinar cells to promote tumorigenesis in mice on HFD	[131]
Ob/ob, db/db	-	GEMM (KC crossed with Ob/ob)	Endocrine-exocrine signaling drives obesity-associated PDAC	[7]
**Prostate cancer**				
DIO	HFD (42% kcal from fat), 4 months	-	HFD promotes prostatic basal-to-luminal differentiation and accelerates intiation of protstate epithelial hyperplasia originated from basal cells	[132]
DIO	HFD (60% kcal from fat), from 3 weeks until endpoint	GEMM (FVB-Tg(ARR2/Pbsn-MYC)7Key/Nci)	High-fat diet fuels prostate cancer progression by rewiring the metabolome and amplifying the MYC program	[117]

CICM – chemical induced cancer model; DIO – diet-induced obesity; GEMM – Genetically engineered mouse model; HFD – high fat diet; T – transplant model; unless otherwise listed, duration of feeding indicates feeding pattern prior to transplantation or induction of cancer (e.g. administration of agents to induce expression of tumor promoters or exposure to carcinogens).

### Genetic animal models

Amongst genetic models, mice with deficient leptin signaling are the most frequently used [8]. In these models, obesity is caused by the lack of leptin (the *ob*/*ob* mouse), or by a mutation in the leptin receptor gene (*db*/*db* mouse), both causing the mice to overfeed. The genetic models consistently induce an early onset obese state and comorbidities such as insulin resistance and hepatic steatosis when the mice are fed standard chow. Their primary disadvantage is the exclusion of potential peripheral effects of leptin on cancer cells and the cells of the tumor microenvironment.

### DIO animal models

The DIO rodent models are established by feeding animals with diets containing a high proportion of fat, sugar or a combination of the two. While several feeding patterns have been developed, the most commonly-used diets contain 30-60% kcals from fat which is provided to the mice for ten to twelve weeks prior to cancer challenge (**Table 1**). The long-term establishment period typically mimics the gradual development of obesity in humans as well as obesity-induced comorbidities that follows human obesity. On the other hand, this model is confounded by the potential biases created by the chosen dietary composition [9]. This could affect tumor outcomes independently of the obese phenotype per se and has led many investigators to include both DIO and genetic models [10–13]. While findings from the two independent model systems generally overlap, opposing results have been reported for intestinal cancer. In this study the authors reported that DIO induced intestinal tumor stemness, the *db/db* system led to a reduction in intestinal stem cell counts [12].

Given that the majority of comorbidities seen in obese individuals are dependent on obesity-induced inflammation [14], the field has relied on syngeneic transplants rather than xenograft models. Major challenges associated with syngeneic models include limited tumor heterogeneity, variable phenotypes depending on injection site, and relatively few transplantable cell lines as exemplified by an extreme reliance on E0771, a murine mammary carcinoma cell line and originally isolated as a spontaneous tumor from C57BL/6 mouse [15] (**Table 1**). Finally, the clinical specificities in the links between obesity and cancer create additional challenges in the model setup. Perhaps the best example of such is the dichotomy between obesity-induced breast cancer in pre- and postmenopausal women. Ovariectomy is a well-established method to induce post-menopausal state in mice [16]. After removal of the ovary, endogenous estrogen production stops and the ovariectomized mice usually develop increased body weight, hepatic steatosis, insulin resistance and impaired glucose tolerance [17]. Subcutaneously implanted tumors grow faster in the ovariectomized model, regardless of diet, and this makes it difficult to distinguish the effects of high-fat diet (HFD) on tumor progression from the effects of postmenopausal loss of hormones [18].

To establish HFD-induced obese mouse models, the feeding period varies from four to 24 weeks, although ten to twelve weeks is commonly used (**Table 1**). This period is sufficient to induce obese phenotypes featuring increased body weight, elevated fasting glucose levels, hyperinsulinemia, insulin resistance, hepatic steatosis, dyslipidemia and hypertension [19]. Such long-term HFD feeding is fairly consistently associated with enhanced tumor initiation and growth. In contrast, shorter HFD feeding periods of just four days is not associated with changes in body weight or features of metabolic syndrome. Such short-term feeding regiments do not affect tumor initiation of orthotopically implanted mouse breast cancer cells [20]. This was evident even though the HFD exposure was maintained for seven weeks until the end of the experiment [20]. Thus, an established state of obesity and obesity-induced physiological changes are required for affecting tumor outcomes. Short feeding periods should thus be avoided [20, 21], and the establishment of obesity-induced clinical and biochemical features should be verified before tumor challenge.

### Reversibility of obesity-induced effects on tumor outcomes

A key question in the field is to what degree reversing the obese phenotype affects tumor outcomes. In the *ob/ob* model, reestablishing leptin expression through adenoviral expression in muscle reversed the obese phenotype and related biochemical parameters, leading to the suppression of pancreatic cancer progression [7]. In the same study, the enhanced tumor progression in the *ob/ob* model was also reversed by dietary caloric restriction. With the DIO models, a number of studies have utilized dietary switch experiments. Five weeks low-fat diet (LFD) feeding after established DIO was enough to reverse obesity-induced inflammation and breast tumor formation [20]. A comparison between a short-term and a long-term dietary switch demonstrated that exposing the DIO mice to LFD for seven days was not sufficient to affect the obesity-induced effects on intestinal stem cells, while the phenotype was reversed after four weeks LFD [12]. Taken together, these observations indicate that obesity-induced effects on tumor outcomes are reversible.

## MOLECULAR MECHANISMS OF THE OBESITY-CANCER CONNECTION

The interaction between obesity and cancer could be governed by genetic as well as non-genetic alterations. Currently there is limited evidence for a genetic component linking obesity to cancer initiation. This likely reflects the difficulty of obtaining body mass index (BMI) measures as well as incorporation of potential cachexia-induced weight loss in large consortia databanks containing patient genomic status. Instead, an expanding number of cellular processes have been suggested to govern obesity effects in malignancies.

### Adipokines affecting carcinogenesis

Adipose tissue is an active endocrine organ which secretes adipokines, chemokines and constituents of the extracellular matrix (ECM) [22]. In response to increased demand of triglyceride storage, adipose tissue undergoes hyperplasia and hypertrophy in the transition from a lean to an obese state. These architectural adjustments induce changes in the adipocyte secretome and released metabolites which contribute to an altered micro- and macroenvironment in the obese compared to the lean setting [22].

#### Leptin

Adipokines have long been hypothesized to provide a mechanistic link between cancer and obesity. Leptin is secreted in proportion to adipose volume and total fat mass [23, 24]. By signaling through leptin receptors in the central nervous system (CNS), leptin regulates energy balance by functioning as a satiety signal, which reduces food intake and increases energy expenditure. In obese individuals, however, the satiety-promoting effects of leptin are impaired by the induction of cellular leptin resistance [25]. Along with decreasing energy expenditure and promoting obesity, hyperleptinemia has pronounced peripheral effects on cancer cells (discussed below) and the tumor microenvironment including immune cells – particularly the T-helper 1 cells [26, 27].

Leptin and the leptin receptors have been identified in malignant cells of various origins including hepatocellular cancer, colorectal cancer, thyroid cancer and breast cancer [28]. Overall, leptin has been assigned a pro-tumorigenic function. In breast cancer, leptin and the leptin receptor are overexpressed in primary and metastatic lesions compared to non-cancer tissues [29, 30]. In cell culture, leptin acts as a growth-stimulating agent for breast cancer cells, promoting proliferation and repressing apoptotic pathways [31]. A similar role has been reported in colon cancer, where leptin acts as a growth factor at stages correlating with tumor initiation in a murine model [32]. Moreover, leptin treatment of cancer cells modulates processes such as metabolic reprogramming [33] and reactive oxygen species production [34, 35].

In females, higher leptin concentrations are associated with increased risk, as well as grade, stage and recurrence of breast cancer [36, 37]. In the obese context, leptin has been linked to tumor-initiating cell survival and obesity-associated triple negative breast cancer development by promoting cancer stem cell enrichment and epithelial-to-mesenchymal transition (EMT) [38, 39]. Leptin governs the stem cell phenotype through epigenetic mechanisms controlled by a leptin-STAT3-G9a histone methyltransferase signaling axis [40].

In sum, several studies support a role of leptin in tumorigenesis which favors cell growth and survival by increasing proliferation and decreasing apoptosis, regulating inflammatory processes, modulating cancer stem cell properties as well as metabolic activity. However, augmented tumor formation is observed for several cancers, including cancers of the liver, kidney and pancreas in *ob/ob* and *db/db* compared with wildtype mice [7, 11, 41, 42] suggesting leptin-independent mechanisms. A number of the early mammary cancer studies were performed with the mouse mammary tumor virus (MMTV)-transforming growth factor-alpha (TGF-α) transgenic mouse model - a cancer model in which TGF-α overexpression is under the control of the MMTV promoter. Without any further genetic alteration, 30-40% of MMTV-TGF-α mice develop spontaneous tumors within 16 months [43]. However, the overexpression of TGF-α in the background of either *ob*/*ob* or *db*/*db* failed to develop tumors, suggesting a required role of leptin signaling in TGF-α driven carcinogenesis [44, 45]. Interestingly, MMTV-Wnt1 cell-derived syngeneic breast cancer cells displayed increased mammary tumor burden upon implantation into *db/db* mice, whereas implantation of the same cells into *ob/ob* mice did not affect tumor growth [38]. This suggests that peripheral cancer cell leptin signaling is required for obesity-dependent effects of MMTV-Wnt1 cells. Taken together, these experiments illustrate that leptin does not solely account for obesity-accelerated tumor progression for all cancer types.

#### Adiponectin

In addition to leptin, deregulation of the adipokine adiponectin has been implicated in driving tumor progression in several obesity-associated cancer types, including colon, liver, breast, renal, gastric, esophageal, pancreatic and endometrial cancer [46]. Circulating adiponectin levels negatively correlate with body fat mass and adiposity. This hormone acts as an insulin sensitizer of tissues such as liver and muscle, as well as balancing glucose and lipid metabolism [47, 48]. Adiponectin mediates its effects through its classical and ubiquitously expressed receptors AdipoR1 and AdipoR2, and the non-classical receptor T-Cadherin [49, 50]. Several signaling pathways are stimulated by adiponectin, including AMPK, PI3K/AKT, MAPK, mTOR, NF-kB and STAT3 [46]. Adiponectin exerts both anti-inflammatory and anti-proliferative effects and is hence often referred to as the “guardian angel adipokine” [48, 51]. In addition, adiponectin also potently stimulates ceramidase activity through AdipoR1 and AdipoR2, and thereby enhance pro-apoptotic ceramide catabolism leading to formation of its downstream anti-apoptotic metabolite sphingosine-1-phosphate (S1P) [52]. HFD is sufficient to induce expression of the enzyme that generates S1P (sphingosine kinase 1, SphK1) and its receptor S1P receptor 1 (S1PR1) in syngeneic and spontaneous breast tumors [53]. Targeting the SphK1/S1P/S1PR1 axis in these models attenuates obesity-induced tumor progression [53].

Epidemiological studies have linked low serum adiponectin levels to an increased risk of colon cancer [54]. This is consistent with *in vivo* studies that have demonstrated promotion of intestinal carcinogenesis by lack of adiponectin in both genetic and chemically induced cancer models [55]. Another study also found a repressing role on colonic epithelial proliferation in a chemically induced cancer model. However, this effect was specific to adiponectin-deficient mice fed an HFD. No effect was observed for adiponectin-deficient mice fed a basal diet compared with wild-type mice [56]. Likewise, adiponectin protects against liver tumorigenesis in nude mice, and its reduced expression is associated with poor prognosis in obese patients with hepatocellular carcinoma [57]. These findings were supported by studies using an orthotopic liver tumor nude mouse model, which showed inhibited tumor growth and lower incidence of lung metastasis in adiponectin treated mice [58]. Furthermore, adiponectin treatment suppressed hepatic stellate cell activation and macrophage infiltration [58]. In addition to anti-inflammatory and growth-suppressing effects, adiponectin has been shown to constrain tumor growth by inhibiting tumor vasculature [58, 59]. On the contrary, lack of adiponectin in a MMTV-polyoma middle T antigen (PyMT) model significantly reduced tumor growth and angiogenesis [60, 61]. Thus, a general role of adiponectin in tumor angiogenesis remains to be defined.

#### Other adipokines

In addition to altered levels of leptin and adiponectin, increased levels of obesity-related adipokines such as interleukin-6 (IL-6), interleukin-8 (IL-8), tumor necrosis factor-α (TNF-α), visfatin and resistin are associated with increased cancer risk [62]. Resistin is mainly expressed in adipocytes and immune cells [63] and has been reported to promote breast tumorigenesis *in vitro* and *in vivo* [64]. In a DIO mouse model, its expression and secretion are significantly increased in mammary adipose tissue. Mechanistically, HFD-induced elevated levels of circulating FFA promote peroxisome proliferator-activated receptor γ (PPARγ) signalling, which is an upstream regulator of WW-domain-containing transcription regulator 1 (WWTR1; also known as TAZ) expression. The enhanced TAZ expression via the FFA/PPARγ axis further activates resistin secretion [64].

Adipose tissue is a major component of the breast. The cytokine secretion from adipocytes is affected by menopausal status, which could link to the paradoxical association of obesity and pre- and post-menopausal breast cancer risk. After menopause, a high estrone (E1): estradiol (E2) ratio in tissue and circulation was observed in both mouse models and human patients. A high intratumor E1:E2 ratio cooperates with NFκB pathway to increase tumour stemness in obesity [65].

In summary, accumulating evidence obtained by a variation of model systems supports an important role of adipokines in tumor progression. On the other hand, knowledge derived from a curious counterpart to the obese phenotype, the fatless A-Zip/F1 mouse, challenges a universal role. The fatless mouse model mimics generalized lipodystrophy and is characterized by the majority of the comorbidities associated with obesity including insulin resistance and ectopic lipid accumulation but lack the secretion of adipokines. In chemical carcinogenesis and transgenic C3(1)/T-Ag mammary models, tumor incidence was consistently higher in the fatless mouse compared with controls - suggesting that adipocyte-independent factors drive tumorigenesis in this environment [66]. Overall, more model-specific research is needed to fully understand the link between obesity, cancer and adipokines.

### Insulin and insulin-like growth factor-1 (IGF-1)

Increased serum concentrations of insulin and IGF-1 are frequently detected in obese individuals. Both factors have been proposed to serve as key hormonal mediators mechanistically linking obesity and cancer [67]. Insulin and IGF-1 are closely related ligands that activate the insulin receptor (INSR) and IGF-1 receptor (IGF-1R), leading to stimulation of two key cell proliferation and protein synthesis pathways for tumorigenesis - the PI3K-AKT-mTOR signaling pathway and the RAS-MAPK pathway (reviewed in [68]). Expression of the respective receptors is detected on cancer cells of different origins and several cancers are driven by insulin and IGF-1 *in vitro* [69–73]. A positive association is also detected between insulin and obesity-prone cancers in terms of tumor growth in rodents as well as stage at diagnosis and death in humans (reviewed in [74]). Moreover, case-control and cohort studies have demonstrated that individuals with higher levels of insulin or C-peptide (indirect insulin measure) are at higher risk for developing obesity-related cancers including breast, endometrial, colorectal, pancreatic, liver, ovarian and gastric cancers compared to individuals with low levels of these factors (reviewed in [75]).

In addition to a mitogenic function in carcinogenesis, insulin has been suggested to effect metabolic processes in cancer cells. Insulin increases mitochondrial glucose oxidation and augments cell division in cells derived from obesity-associated tumors, including colon, breast and prostate cancer. In contrast, no alteration of substrate preference is observed in obesity-independent cell lines (melanoma, lymphoma and small cell lung cancer) [76]. In studies using an insulin lowering agent, dapagliflozin, a sodium-glucose cotransporter-2 (SGLT2) inhibitor, reduction in insulin levels slowed obesity-accelerated tumor growth of both syngeneic breast and colon cancer models. The authors concluded that this effect is not due to increases in ketosis or to a direct effect on tumor cell division, but rather is mediated by the reversal of hyperinsulinemia, resulting in diminished tumor glucose uptake and oxidation [77]. At present, several human clinical trials are investigating insulin-lowering treatments as adjuvants to cancer treatments (reviewed in [74]).

### ECM remodeling and fibrosis

Tumors are composed of cancer cells along with their connected stromal compartment. The tumor stroma consists of vasculature, non-transformed cell types including fibroblasts and immune cells, as well as structural elements such as the basement membrane and ECM. The stromal compartment is an integral part of cancer initiation, growth and progression (reviewed in [78]) and a number of studies have begun to delineate how obesity-induced stromal alterations influence tumor growth. Adipose tissue in obese individuals is among others characterized by an altered biochemical as well as biophysical microenvironment [79]. For instance, adipocytes in expanding adipose tissue deposit altered amounts of ECM components causing ECM remodeling and changes in tissue stiffness. Furthermore, obese tissue ECM contains more aligned fibers and higher interstitial pressure (reviewed in [80]). These changes are not restricted to subcutaneous and visceral fat but occur also in other fat depots. For instance, the homeostasis of mammary fat is disrupted in obese mouse models, as this tissue is enriched in myofibroblasts and stiffness-promoting ECM components [81]. These alterations, which are characteristic features of desmoplasia and fibrosis, can promote the tumorigenic potential of premalignant human breast epithelial cells [81]. Research on mouse models has shown that obesity also promotes desmoplasia in the pancreas, a common histological feature of PDAC, which is associated with accelerated tumor growth and impaired delivery/efficacy of chemotherapeutics through reduced perfusion [42]. Interestingly, it was suggested that the exacerbated desmoplasia and augmented tumor growth was a result of crosstalk between adipocytes, tumor-associated neutrophils and pancreatic stellate cells [42]. Grohmann *et al.* reported how obesity-associated hepatic stress could independently contribute to the pathogenesis of non-alcoholic steatohepatitis (NASH), fibrosis and hepatocellular carcinoma (HCC). Obesity promotes hepatic STAT1 dependent T-cell infiltration, NASH and fibrosis as well as NASH-independent STAT3-dependent HCC [82].

Moreover, single ECM components that are deregulated in the obese state have been demonstrated to promote tumorigenesis. One example is collagen VI, which is abundantly produced and secreted by adipocytes. A recent study applying proteomic analysis of ECM isolated by *in vitro* decellularization methods identified collagen VI to be up-regulated in murine obese mammary gland and breast tumor tissues relative to lean tissues [83]. Adipocyte-derived collagen VI has previously been demonstrated to be important for early mammary tumor progression, as collagen knockout mice in the background of the MMTV-PyMT mammary cancer model have reduced rates of early hyperplasia and primary tumor growth [84]. *In vitro* studies point to that full-length collagen VI is a driver of triple negative breast cancer cell adhesion, migration and invasion [83].

Furthermore, obesity-associated ECM remodeling has been shown to regulate the properties of immune cells. Culturing of bone-marrow-derived macrophages on ECM isolated from obese mice induce proliferation, changes the morphology, impact polarization as well as promote angiogenic traits compared to cells cultured on top of ECM derived from lean mice [85]. Hermano *et al.* reported that macrophage function could be regulated by heparanase, an endoglucuronidase that cleaves heparin sulfate in ECM [86]. They provided evidence that heparanase preferently is expressed in clinical/experimental obesity-associated breast tumors, and that heparanase deficiency abolishes obesity-accelerated orthotopic tumor growth. In the obese environment, they showed that heparanase stimulates macrophage production of inflammatory mediators that induce aromatase, a rate-limiting enzyme in estrogen synthesis [86].

### Cancer metabolism

Metabolic reprogramming is commonly observed in cancer cells during tumor progression. Given the multiple local and systemic metabolic abnormalities in obese individuals, the obese state provides an interesting case for new discoveries in the interface between systemic and tumor metabolism.

#### The effects of FFA on tumor initiation

Recent studies have proposed that lipid metabolism and fatty acid oxidation contribute to cancer stemness. A subpopulation of human carcinoma cells that expressed high levels of the fatty acid transporter CD36 and lipid metabolism genes was identified to possess cancer stemness features and to contribute to a poor prognosis in human cancer patients [87, 88]. In intestinal cancers, exposure to FFA was demonstrated to activate PPARδ-dependent signaling to endow tumor-initiating capacity [12]. In addition to direct cancer cell-autonomous effects, obesity-induced elevated levels of saturated fatty acid drive the accumulation of metabolically activated macrophages in adipose tissue. Such macrophages were shown to enhance stem-like properties in triple negative breast cancer cells and promote tumor initiation in obese mouse models [20]. Overall, there is compelling evidence that fatty acids could be a critical link between obesity and tumor initiation.

#### The effects of FFA on tumor progression

In addition to enhanced tumor initiation, emerging evidence indicates that the elevated circulating FFA and fatty acid binding proteins in the obese state are associated with cancer progression. In ovarian cancer, the rapid growth and metastatic colonization of cancer cells were suggested to be directly fueled by fatty acids delivered by fatty acid binding protein 4 (FABP4), also known as adipocyte FABP (A-FABP), from adipocytes [89]. Furthermore, lipolysis in adipocytes and increased β-oxidation rate in cancer cells were observed in co-culture experiments, suggesting that adipocyte-derived fatty acids can act as an energy source fueling cancer cells [89]. Hao *et al.* expanded the role of FABP itself. They demonstrated that increased circulating levels of A-FABP enhanced breast cancer stemness and aggressiveness in both *in vitro* and *in vivo* models [90]. Moreover, the authors further determined that the association is dependent on STAT3/ALDH1 signaling regardless of endogenous A-FABP level, suggesting a dual mechanism by which A-FABP supports tumor progression both as a fatty acid transporter and as a signaling molecule [90]. Consistently, Madak-Erdogan *et al.* demonstrated that obesity-induced elevated circulating FFA could be taken up by estrogen-positive breast cancer cells and promote cancer proliferation in obese postmenopausal women [91]. Also, these findings suggested that fatty acids activate a mTOR and MAPK dependent signaling network to facilitate increased glycolytic and aerobic respiration in breast cancer cells. Finally, cancer cells and immune cells display distinct metabolic adaptations in the obesogenic tumor microenvironment [92]. For example, HFD represses PHD3 expression specifically in cancer cells, which rewire their metabolism to accelerate fatty acid uptake and oxidation. This in turn impacts the fatty acid availability for T cells in the same microenvironment and thereby impairs T cells infiltration and function [92].

#### Other metabolites and tumor progression

In addition to fatty acid-related adaptations, modifications in other metabolic pathways have been identified in tumors evolving in obese environments, including glucose handling, nitrogen metabolism and pyruvate-dependent mitochondrial respiration. For example, in a genetic model for pancreas cancer, HFD feeding heightened aerobic glycolysis through hyperactivation of oncogenic KRAS [93]. Moreover, transcriptomic analysis of an orthotopic xenograft PDAC model revealed an enrichment of nitrogen metabolism pathways [94]. The authors identified the mitochondrial enzyme arginase 2 (ARG2), which catabolizes arginine into ornithine and urea, to be induced in obese mouse tumors and that its expression level correlated with patient BMI. As an important enzyme at the final step of the urea cycle, the obesity-induced upregulated ARG2 enhances nitrogen flux into the urea cycle. Moreover, the depletion of ARG2 causes ammonia accumulation and suppresses PDAC, particularly in obese hosts [94]. In a search for mechanisms underlying the increased cancer risk that is associated with the combination of metabolic deregulation and circadian disruption, Ramos *et al.* demonstrated that a non-canonical function of BMAL1 limits obesity-promoted triple-negative breast cancer [95]. BMAL1, a key circadian transcription factor, suppresses the flexibility of mitochondrial substrate usage and pyruvate-dependent mitochondrial respiration induced by chronic insulin treatment *in vitro*. Interestingly, orthotopic transplantation of E0771 breast cancer cells depleted for BMAL1 revealed that BMAL1 functions as a tumor suppressor in obese, but not in lean mice. In humans, down-regulation of BMAL1 is associated with higher risk of metastasis [95].

### Inflammation

Obesity is associated with local and systemic immune system dysregulation characterized by increased secretion of inflammatory cytokines and phenotypic conversions in immune cells [96]. Recently, a growing number of studies have suggested that obesity-associated inflammatory alterations might promote progression of multiple cancer types through diverse mechanisms.

#### Tumor immune cell abundances

A fundamental question is whether obesity-induced chronic inflammation alters immune cell infiltration in tumors. At the histological level, HFD-feeding increases infiltration of tumor-associated macrophages in both transplant and some autochthonous PDAC models [97–99] as well as in transplant models of breast cancer [53, 86] and in chemically induced hepatocarcinoma [100, 101]. In contrast, transcriptional analysis of the duodenum of K-rasG12Dint mice revealed down regulated F4/80 expression in mice fed HFD [102], and fluorescence-activated cell sorting (FACS) analysis of transplanted E0771 breast tumors showed a reduction of CD11b^+^F4/80^+^ macrophages [103]. In kidney cancer, increased numbers of inhibitory dendritic cells (DC) infiltrate tumors of DIO mice [104]. Similar observations have been reported in breast cancer, as a population of CD11b^+^F4/80^−^ cells (consisting of neutrophils and monocytes) identified by FACS showed a 31% increase in E0771 tumors from HFD mice [103]. Taken together, conflicting results on obesity-induced immune cell tumor infiltration has been reported for different cancer types as well as for different cancer/obesity models applied. This is in line with our own preliminary findings. We interrogated the immune-infiltrating cells in tumors grown in obese and non-obese mouse models using a 36-marker immune-focused mass cytometry panel [105]. Immunotyping of three syngeneic breast cancer models and two pancreas cancer models revealed that tumor immune infiltrate composition is highly model- and cancer type-specific. While no major immune cell alterations were observed in the pancreas cancer models and in two of the breast cancer models, HFD-feeding increased two T cell suppressive cell types and decreased CD8 T-cells in the E0771 breast cancer model [105].

#### Macrophages

In addition to affecting the overall infiltration of immune cells, accumulating evidence indicates that obesity affects immune function through modulating immune cell phenotypes. The immune subset that has so far received the most attention is macrophages. In several cancer models, obesity has been proposed to regulate recruitment, polarization and signaling of macrophages, thereby contributing to accelerated tumor progression. In breast and pancreatic cancer models, obesity induces recruitment of tumor-associated macrophage (TAM) with an M2-like cytokine profile. VEGFR-1 is abundantly expressed in TAMs, and blockade of VEGFR-1 signaling in obese but not lean mice leads to a shift in pro-tumor cytokine production and TAM polarization from an M2 pro-tumor to an M1 anti-tumor phenotype that ultimately reduce obesity-induced tumor progression [97]. Furthermore, the elevated level of fatty acids during obesity drives polarization of adipose tissue macrophages towards a metabolically activated phenotype. This phenotypic shift alters the niche to support triple negative breast cancer stemness and tumorigenesis through an IL-6/GP130 signaling axis [20]. In colitis-associated colorectal cancer, signaling through obesity-induced IL-6 was reported to shift macrophage polarization towards a tumor-promoting phenotype that produced the chemokine CC-chemokine-ligand-20 (CCL-20) in the tumor microenvironment. CCL-20 in turn, promoted tumorigenesis by recruiting CC-chemokine-receptor-6 (CCR-6)-expressing γδ T and B cells via chemotaxis [106]. Obesity-accelerated cancer growth is also associated with increased IL-6 levels and shift in M2/M1 macrophage ratio in a genetic mouse model of prostate cancer. Interestingly, these effects could specifically be blocked in HFD-fed mice by inhibition of the IL-6 receptor [107].

In two syngeneic orthotopic transplant models, a NLRC4 inflammasome/IL-1β signaling was demonstrated to provide a link between obesity and breast cancer progression. The obese tumor microenvironment induced an increase in the number of tumor-infiltrating macrophages with an activated NLRC4 inflammasome, which further activated IL-1β. The NLRC4/IL-1β module was able to promote angiogenesis via up-regulation of VEGFA, which in turn to contributed tumor progression [108]. In a liver cancer model, HFD induced liver endoplasmic reticulum (ER) stress boosted macrophages-mediated production of inflammatory cytokines. This in turn activated TNF receptor 1 (TNFR1)-IκB kinase β (IKKβ) signaling, which thereby contributed to hepatocyte proliferation and formation of HCC [101]. The importance of macrophages in the connection between obesity and breast cancer has however been challenged. Bousquenaud *et al.* reported that HFD tumors contained reduced amount of M1 macrophages, and that depletion of macrophages by clodronate liposomes boosted tumor growth in HFD mice. Hence, the authors concluded that macrophages do not contribute to promote obesity accelerated tumor progression [103].

#### T cells

Tumoral T cell function is of great interest, especially since the T cell is the critical mediator of immunotherapies, including adoptive T cell therapy and immune checkpoint therapy. Obese cancer patients have been reported to display increased levels of dysfunctional and exhausted T cells [92, 109], suggesting that immunotherapy could be a promising treatment option for obese cancer patients. Indeed, in both human patients and preclinical animal models, obesity associates with better response to anti-PD1 and anti-CD8 immune checkpoint therapy [92, 109, 110].

#### Blocking obesity-induced inflammation

To address whether inflammatory processes are required for obesity-induced tumor initiation and progression, several studies have obstructed inflammatory signaling pathways, through genetic depletion of key cytokines or pharmacological inhibition of specific immune cell populations in animal models. Systemic knockout of TNF-α, IL-6 or TNFR1 all prevent obesity-induced cancer formation in HCC [100] and PDAC [99] suggesting a functional role of the immune apparatus. Although these studies are limited by the systemic depletion, they indicate a required role of the immune system in obesity-induced cancers.

Experimental evidence across cancer types supports a pivotal role of cyclooxygenase-2 (COX-2)-mediated inflammatory pathways in obesity promoted cancer progression [98, 111]. A study applying an adult acinar-specific mutant Kras PDAC model, demonstrated that high fat consumption can activate and sustain Kras activity via COX-2 induction and COX-2 mediated positive feedback loops, which in turn contribute to the development of PanINs and PDAC. Inhibition and genetic knockout of COX-2 prevented pancreatic inflammation and initiation of PDAC, suggesting important roles for COX-2-dependent inflammatory processes [98]. In contrast, treatment with aspirin, a nonsteroidal anti-inflammatory agent (NSAID) that blocks the cyclooxygenase enzymes, did not impact tumor progression, immune cell infiltration or fibrosis in an obese genetic model of PDAC [7]. Consistently, the growth advantage of orthotopically implanted oncogenic *Kras*-driven PDAC cell lines in the obese environment was sustained in obese C57Bl6 mice lacking T-, B- and NK-cells (*Rag2*^-/-^::*CD47*^-/-^::*Il2rg*^-/-^) [105].

### Microbiome

An integrated part of the obese phenotype is alterations in the microbiota composition [112, 113]. As a natural part of the tumor microenvironment of gastrointestinal malignancies, the altered microbiota can directly participate in the regulation of gastrointestinal cancer progression. In addition to such local effects, microbiota-mediated metabolites display both systemic metabolic and inflammatory changes [114, 115]. Gut microbiota are the major producers of short-chain fatty acids (SCFAs), which can be the utilized as an energy source and as key signaling molecules. Feeding mice with an HFD results in the decreased production of multiple SCFAs including acetate, propionate and butyrate and were demonstrated to promote G12D mutant Kras-driven intestinal carcinogenesis [102]. Interestingly, butyrate supplement through feeding successfully reversed the cancer progression effect of the high-fat feeding. Also, fecal-transplantation from HFD-fed to normal diet fed Kras mutant mice was sufficient to induce tumorigenesis [102]. These findings suggest that an HFD-induced microbiota shift synergizes with the Kras mutation during tumorigenesis and that such effects could act independently of obesity. In chemically-induced HCC, obesity-dependent alterations of the gut microbiota promote tumorigenesis through deoxycholic acid - a secondary bile acid [11, 111]. This metabolite could cause DNA damage and provoke a senescence-associated secretory phenotype (SASP) in hepatic stellate cells to promote HCC development. In addition to bile acids, the altered gut microbiota provided lipoteichoic acid to the liver, and this compound synergistically promoted the SASP in hepatic stellate cells. Also, lipoteichoic acid is able to induce the COX-2-mediated immunosuppression in liver immune cells [111]. Altogether, the microbiota is an important component when studying gastrointestinal cancers in obesity. Due to the complex environment, further studies are required to understand and establish a solid mechanistic basis of the relationships between the microbiome, obesity and gastrointestinal cancers progression.

## SUMMARY AND FUTURE PERSPECTIVES

As an expanding number of reports aiming to identify molecular connections between the obese state and tumors are released, it is clear that the complexity of the obese environment likely translates into integrated and complex molecular drivers. This necessitates the use of proper model systems that realistically recapitulates such environments. Whereas we have so far relied on mouse models, additional model systems as for example organoids grown in obese and non-obese conditions could become valuable alternatives to alleviate the limitations linked to mouse studies described above.

So far, the most consistent molecular link between the obesogenic environment and tumorigenesis revolves around increased abundance of fatty acids, ECM remodeling and immune cell differentiation. Fatty acids have been implicated in both tumor growth and tumor initiation through cell intrinsic and extrinsic mechanisms. In the tumor cells FFA have been suggested to act as an alternative fuel source while FFA have also been shown to modulate metabolically-activated macrophages and T-cells to create a pro-tumorigenic microenvironment. Direct effects of the obese environment on T-cells further translate into a potential added benefit of immune checkpoint therapy in the obese setting. Through tumor promoting modulation of the stromal compartment of the microenvironment, obesity leads to both biophysical changes driven by alterations in myofibroblast cell populations and biochemically through altered collagen VI secretion and processing.

So far, the vast majority of mechanistic studies have focused on obesity-dependent alterations in adipocytes, immune cells and their secreted molecules. However, recent work urges us to take a broader look at additional cell types that could be affected by the obese state. This, for example, include the islet cells of the pancreas. In the obese setting, these adapt by inducing Cck which in turn acts locally on pancreatic acinar to cells to drive tumorigenesis [7].

Another area of increasing interest is the connection between obesity and epigenetic deregulation. This connection was previously demonstrated in somatic tissues and has recently also been investigated in tumors [116]. Epigenetic rearrangements in cancers grown in obese mice, have been reported in prostate cancer [117], colorectal cancer [10] and breast cancer [118]. How the obese environment induces such changes is currently unknown, but it is clear that it could have profound effects on tumorigenesis. Interestingly, many metabolic intermediates including Acetyl-CoA and S-adenosyl methionine act as epigenetic precursors and can directly and indirectly affect chromatin modifications and function [119]. Going forward, it will be interesting to investigate if obesity-dependent changes in cellular metabolites are potential drivers of chromatin remodeling in tumors.

While the epidemiological link between obesity and cancer risk is well proven, the link between obesity and metastatic spread is less clear. This is reflected in far fewer mouse studies of metastasis in the obese setting. In one study, breast cancer lung metastatic colonization was enhanced in obese mice via GM-CSF- and IL-5 dependent recruitment of neutrophils [13]. Further studies in breast cancer using transplant and genetic models suggest that obesity-dependent changes in the sphingolipid profile is driving metastatic colonization in the obese setting [53]. However, potential mechanisms of obesity linked metastatic spread are currently understudied.

Finally, in addition to potentially revealing new exciting mechanistic pathways, discoveries of dependencies of the obesity-cancer link might lead to the development of novel targeted therapies for the benefit of growing group of obese cancer patients.

## Abbreviatons:

ARG2: – arginase 2;
BMI: – body mass index;
COX2: – cyclooxygenase-2;
DIO: – diet-induced obesity;
ECM: – extracellular matrix;
FABP: – fatty acid binding protein;
FACS: – fluorescence-activated cell sorting;
FFA: – free fatty acid;
HCC: – hepatocellular carcinoma;
HFD: – high-fat diet;
IGF: – insuline growth factor;
IL: – interleukin;
LFD: – low-fat diet;
MMTV: – mouse mammary tumor virus;
NASH: – non-alcoholic steatohepatitis;
PDAC: – pancreatic ductal adenocarcinoma;
PPARγ: – peroxisome proliferator-activated receptor γ;
PyMT: – polyoma middle T antigen;
S1P: – sphingosine-1-phosphate;
SCFA: – short-chain fatty acid;
TGFα: – transforming growth factor-alpha;
TNFα: – tumor necrosis factor-α.
